# Impact of type 2 diabetes mellitus on mid-term mortality for hypertrophic cardiomyopathy patients who underwent septal myectomy

**DOI:** 10.1186/s12933-020-01036-1

**Published:** 2020-05-13

**Authors:** Shengwei Wang, Hao Cui, Keshan Ji, Changpeng Song, Changwei Ren, Hongchang Guo, Changsheng Zhu, Shuiyun Wang, Yongqiang Lai

**Affiliations:** 1 Department of Cardiovascular Surgery Center, Beijing Anzhen Hospital, Capital Medical University, Beijing Institute of Heart, Lung and Blood Vascular Diseases, No. 2, Anzhen Road, Chaoyang District, Beijing, 100029 China; 2 Department of Cardiovascular Surgery, Mayo Clinic, Rochester, MN USA; 3 Department of Special Medical Treatment Center, State Key Laboratory of Cardiovascular Disease, Fuwai Hospital, National Center for Cardiovascular Diseases, Chinese Academy of Medical Sciences and Peking Union Medical College, Beijing, China; 4 Department of Cardiovascular Surgery, State Key Laboratory of Cardiovascular Disease, Fuwai Hospital, National Center for Cardiovascular Diseases, Chinese Academy of Medical Sciences and Peking Union Medical College, No. 167, Beilishi Road, Xicheng District, Beijing, 100037 China

**Keywords:** Type 2 diabetes mellitus, Hypertrophic cardiomyopathy, Septal myectomy, Sudden cardiac death, Cardiovascular death

## Abstract

**Background:**

Type 2 diabetes mellitus is common in cardiovascular disease. It is associated with adverse clinical outcomes for patients who had undergone coronary artery bypass and valve operations. The aim of this study was to evaluate the impact of type 2 diabetes mellitus on the midterm outcomes of patients with hypertrophic cardiomyopathy who underwent septal myectomy.

**Methods:**

We retrospectively analyzed the data of 67 hypertrophic cardiomyopathy patients with type 2 diabetes mellitus who underwent septal myectomy from two medical centers in China from 2011 to 2018. A propensity score–matched cohort of 134 patients without type 2 diabetes mellitus was also analyzed.

**Results:**

During a median follow-up of 28.0 (interquartile range: 13.0–3.0) months, 9 patients died. The cause of death of all of these patients was cardiovascular, particularly sudden cardiac death in 3 patients. Patients with type 2 diabetes mellitus had a higher rate of sudden cardiac death (4.5% vs. 0.0%, p = 0.04). The Kaplan–Meier survival analysis revealed that the rates of predicted 3-year survival free from cardiovascular death (98.1% vs. 95.1%, p = 0.14) were similar between the two groups. However, the rates of predicted 3-year survival free from sudden cardiac death (100% vs. 96.7%, p = 0.01) were significantly higher in hypertrophic cardiomyopathy patients without type 2 diabetes mellitus than in those with type 2 diabetes mellitus. Furthermore, after adjustment for age and sex, only N-terminal pro-brain natriuretic peptide (hazards ratio: 1.002, 95% confidence interval: 1.000–1.005, p = 0.02) and glomerular filtration rate ≤ 80 ml/min (hazards ratio: 3.23, 95% confidence interval: 1.34–7.24, p = 0.047) were independent risk factors for hypertrophic cardiomyopathy patients with type 2 diabetes mellitus.

**Conclusions:**

Hypertrophic cardiomyopathy patients with and without type 2 diabetes mellitus have similar 3-year cardiovascular mortality after septal myectomy. However, type 2 diabetes mellitus is associated with higher sudden cardiac death rate in these patients. In addition, N-terminal pro-brain natriuretic peptide and glomerular filtration rate ≤ 80 ml/min were independent risk factors among hypertrophic cardiomyopathy patients with type 2 diabetes mellitus.

## Background

Hypertrophic cardiomyopathy (HCM) is a disease state characterized by unexplained left ventricular (LV) hypertrophy associated with nondilated ventricular chambers in the absence of other cardiac or systemic diseases. It is considered a common inherited heart disease and has a population prevalence of 1 in 500 [1, 2]. Approximately two-thirds of patients have left ventricular outflow tract (LVOT) obstruction known as hypertrophic obstructive cardiomyopathy (HOCM). If the symptoms of these patients are refractory to optimal medical therapy, surgery is recommended.

Diabetes mellitus (DM) is highly common among patients with cardiovascular disease and is associated with increased burden of morbidity and hospitalization [3]. The functional changes occurring in DM can significantly alter the hemodynamic stress on the heart. It is an established risk factor for cardiovascular disease and increases the risk of cardiovascular mortality [4]. In addition, previous studies have reported that type 2 DM (T2DM) is associated with adverse cardiovascular events after coronary artery bypass and valve operations [5–7]. However, the effect of T2DM on clinical outcomes for HCM patients who had undergone septal myectomy is not well studied. We aimed to evaluate the effect of T2DM on the clinical outcomes of patients who underwent septal myectomy.

## Methods

### Aim, design, and setting

The objective of this study was to evaluate the effect of T2DM on the clinical outcomes of patients who underwent septal myectomy. This retrospective study was performed using data from two medical centers in Beijing, China.

### Study population

We retrospectively studied the data of 67 patients with HOCM and T2DM from Fuwai Hospital (47 patients) and Anzhen Hospital (20 patients) between 2011 and 2018. A control group (patients without DM) was generated from the two centers and the patients were matched in a ratio of 2:1 based on age, sex, body mass index (BMI), and prevalence of hypertension and hyperlipemia. These patients were randomly selected from subjects who underwent septal myectomy during the same period. The diagnostic criteria of HCM mainly included an unexplained septal hypertrophy with a thickness more than 15 mm according to guidelines [1, 2]. The indications for septal myectomy were severe symptoms or syncope or near-syncope despite optimal medical therapy and LVOT gradient > 50 mmHg at rest or with provocation. The diagnosis of DM was obtained from the clinical chart at the time of evaluation. The details of the surgical methods were described in our previous studies [8, 9]. Concomitant procedures were performed according to the results of the preoperative evaluation and intraoperative exploration. History of non-sustained ventricular tachycardia (NSVT; defined as an episode of consecutive ventricular beats with a rate of at least 100 bpm and a maximum episode length of 30 s) and atrial fibrillation (AF) was recorded, based on history, electrocardiograms, and Holter monitoring in all patients.

### Echocardiography and cardiovascular magnetic resonance imaging

Echocardiographic examinations were performed on patients using an E9 ultrasound system. The thicknesses of the interventricular septum and ventricular wall were determined during diastole. Aside from the maximum thickness, the representative interventricular septal thickness, which is usually the thickness of the point 25 mm under the right coronary sinus nadir, was also recorded to indicate overall thickness. The LVOT gradient was calculated using the simplified Bernoulli equation. Pulmonary hypertension was defined as a pulmonary artery systolic pressure ≥ 35 mmHg. The measurements of left ventricular ejection fraction (LVEF) were determined by following the American Society of Echocardiography recommendations [10].

Cardiovascular magnetic resonance (CMR) examinations were performed using a 1.5-T MR scanner (Magnetom Avanto; Siemens Medical Solutions, Erlangen, Germany). Cine scans in cardiac short- and long-axis views were acquired by applying true imaging with steady-stage precession sequence (TrueFISP). Image analysis using a commercial imaging workstation (Siemens Medical Systems). LVEF and indexed LV mass and volume were measured by analyzing the short-axis cine image. The inner and outer myocardial edges were manually delineated. Late gadolinium enhancement (LGE) was determined semi-automatically as a percentage of the total myocardium and defined as having an intensity > 6 standard deviation above the normal myocardium according to a previous study [11]. Right ventricular ejection fraction (RVEF) was measured using volumetric measurements on CMR [12].

### Laboratory measurements

A fasting blood sample was obtained from all patients on the second day of hospitalization. The levels of high-sensitivity C-reactive protein (hs-CRP), creatinine, low-density lipoprotein, and high-density lipoprotein were monitored. We estimated the glomerular filtration rate using the CKD-EPI equation: a × (serum creatinine/b)c × (0.993)age, where a was 144 and 141 for women and men, respectively; b was 0.7 and 0.9 for women and men, respectively; c was − 0.329 and − 1.209 if the creatinine level was < 0.7 and > 0.7 mg/dL, respectively, for women, and − 0.411 and − 1.209 if the creatinine level was < 0.7 mg/dL and > 0.7 mg/dL, respectively, for men.

### Follow-up data

Clinical status of the study patients was obtained through telephone interviews at least once a year after septal myectomy. Those subjects who died were treated as the endpoints, and the follow-up time was defined as their dead time. The last follow-up of survivors was conducted in December 2018. The causes of death were sudden cardiac death (SCD), death related to congestive heart failure and other cardiovascular disease, or noncardiac death. Because there were no noncardiac deaths in these patients, the survival analysis used in the present study only included cardiovascular death and SCD.

### Statistical analysis

In the present study, we performed a propensity score match for the main variables found to differ significantly (p < 0.05) according to diabetes status: age, sex, BMI, hypertension, and hyperlipemia. Matching was performed using the nearest neighbour method, assigning patients with diabetes and without diabetes in a 1:2 ratio, with a 0.2 caliper width. The equalization test after matching are shown in Additional file 1: Table S1 and Additional file 2: Figure S1.

Results are presented as mean ± standard deviation, median (interquartile range [IQR]), or percentage, as appropriate. Student t-test and Mann–Whitney U test for matched samples were used to compare continuous variables, and the χ^2^ and Fisher’s exact test were used to compare classification variables, as appropriate. The Kaplan–Meier method was used to calculate survival free from cardiovascular death and compare SCD between the two groups. A log-rank test was used to compare survival curves between the two groups. A stepwise multiple Cox analysis technique was used to identify the variables independently associated with cardiovascular death in these patients which were incorporated into the final models. Age, sex, and variables with p < 0.1 on the univariable analysis were added to the multivariable analysis. All reported probability values were two-tailed, and p < 0.05 was considered statistically significant. SPSS version 26.0 statistical software (IBM Corp., Armonk, NY, USA) and R version 3.5.0 (R Foundation for Statistical Computing, Vienna, Austria) were used for calculations and illustrations in the present study.

## Results

### Preoperative and perioperative patient characteristics

A total of 201 HOCM patients (with T2DM, 67 patients; without T2DM, 134) were included. We compared the baseline characteristics between the two groups (Table 1). Compared with patients without DM, those with HOCM and DM had a lower glomerular filtration rate and hs-CRP level. There was no difference in symptoms, including chest pain, palpitation, and syncope, between the two groups. In addition, the prevalence of AF was significantly higher in patients with HOCM and T2DM than in those with HOCM alone. Of the 67 patients with T2DM, 19 (28.4%) were treated with insulin, 39 (58.2%) were treated with metformin, 9 (13.4%) patients were treated with acarbose, and 3 (4.5) patients were treated with metformin and acarbose. In addition, the baseline characteristics of patients with and without T2DM before matching are shown in Additional file 3: Table S2.

**Table 1:** Baseline patient characters

Variable	No diabetes (n = 134)	Diabetes (n = 67)	p value
Age, years	48.8 ± 13.5	50.1 ± 13.8	0.53
Male, n	78 (58.2%)	41 (61.2%)	0.69
Body mass index, kg/m^2^	25.9 ± 3.9	25.6 ± 3.7	0.69
Family history of HCM or SCD, n	18 (13.4%)	15 (22.4%)	0.11
Heart rate, beats/min	72.2 ± 9.3	72.7 ± 8.5	0.71
BNP, pg/mL	1379.9 (636.3–2342.4)	1605.5 (573.7–2726.3)	0.71
Creatinine, umol/L	77.3 ± 13.4	76.8 ± 14.8	0.83
Glomerular filtration rate, ml/min	99.8 ± 20.2	93.7 ± 18.5	0.04
Hs-CRP, mg/L	0.97 (0.40–1.80)	1.30 (0.56–2.28)	0.04
LDL, mmol/L	2.6 ± 0.9	2.5 ± 0.6	0.31
HDL, mmol/L	1.2 ± 0.5	1.16 ± 0.3	0.63
Comorbidities			
Hypertension, n	54 (40.3%)	28 (41.8%)	0.84
Hyperlipemia, n	28 (20.9%)	15 (22.4%)	0.81
Clinical presentation			
Chest pain, n	35 (26.1%)	20 (29.9%)	0.58
Palpitation, n	11 (8.2%)	8 (11.9%)	0.39
Syncope, n	14 (10.4%)	8 (11.9%)	0.75
Atrial fibrillation, n	16 (11.9%)	19 (28.4%)	0.004
Echocardiographic indices			
LVEDD, mm	41.8 ± 5.7	41.5 ± 4.5	0.70
IVST, mm	20.3 ± 5.6	20.0 ± 5.8	0.74
Posterior wall, mm	11.9 ± 2.4	12.2 ± 2.9	0.56
LVEF, %	71.4 ± 5.6	71.3 ± 5.4	0.94
IVST ≥ 30 mm, n	11 (8.2%)	4 (6.0%)	0.57
Moderate or severe MR	24 (17.9%)	7 (10.4%)	0.17
Medical therapy			
Beta-blockers, n	99 (73.9%)	50 (74.6%)	0.91
Calcium-channel blockers, n	7 (5.2%)	7 (10.4%)	0.17
ACEI/ARB, n	17 (12.7%)	8 (11.9%)	0.88
Statins, n	14 (10.4%)	11 (16.4%)	0.23
Insulin, n	–	19 (28.4%)	–
Metformin, n	–	39 (58.2%)	–
Acarbose, n	–	12 (17.9%)	–

Values are presented as percentage, mean ± SD, or median (interquartile range) when appropriate

*IVST* interventricular septal thickness, *HCM* hypertrophic myocardiopathy; *SCD* sudden cardiac death, *NYHA* New York Heart Association, *BNP* brain natriuretic peptide, *LVEF* left ventricular ejection fraction, *LDL* low density lipoprotein, *HDL* high density lipoprotein, *LVEDD* left ventricular end diastole diameter, *LVOT* left ventricular outflow tract, *MR* mitral regurgitation, *ACEI/ARB* angiotensin-converting enzyme inhibitor or angiotensin receptor blocker

The perioperative period was 30 days after the operation. Patients with HCM and T2DM had a higher proportion of coronary artery bypass and maze procedures. The immediate postoperative LVOT gradient was higher in patients with HCM and T2DM. Only one patient died during the perioperative period in the T2DM group (0% vs. 1.5%, p = 0.33). However, the other parameters did not differ between the two groups during the perioperative period. Detailed information is shown in Table 2.

**Table 2:** Perioperative data between two groups

Variable	No diabetes (n = 134)	Diabetes (n = 67)	p value
Concomitant procedures			
Mitral valve procedure, n	22 (16.4%)	9 (13.4%)	0.58
Tricuspid valvuloplasty, n	15 (11.2%^)	7 (10.4%)	0.87
CABG, n	6 (4.5%)	8 (11.9%)	0.05
Maze procedure, n	5 (3.7%)	8 (11.9%)	0.03
Cardiopulmonary bypass time, min	107.6 ± 47.1	113.3 ± 51.2	0.43
Aortic crossclamping time, min	72.7 ± 36.4	76.8 ± 35.9	0.45
Postoperative ventilation time, min	17.0 (13.0–21.0)	17.0 (13.0–24.0)	0.98
Postoperative hospital stays, day	8.4 ± 3.8	8.4 ± 4.2	0.96
Post-operative LVOT gradient, mmHg	6.6 ± 2.7	7.6 ± 3.4	0.04
Perioperative death	0 (0.0%)	1 (1.5%)	0.33

Values are presented as percentage, mean ± SD, or median (interquartile range) when appropriate

*CABG* coronary artery bypass graft, *ICU* intensive care unit

### CMR findings

In the present study, 150 patients underwent CMR. As shown in Table 3, the RVEF was higher in patients without T2DM, whereas the other parameters, including the percentage of LGE, did not differ between the two groups.

**Table 3:** Parameters of cardiac magnetic resonance

Variable	No diabetes (n = 98)	Diabetes (n = 52)	p value
RVEF, %	47.5 ± 7.8	43.9 ± 8.6	0.01
Maximal ISVT, mm	22.2 ± 5.3	22.1 ± 5.1	0.91
Indexed LV mass, g/m^2^	99.1 ± 36.2	92.3 ± 38.1	0.28
Indexed LV volume, ml/m^2^	93.7 ± 36.9	87.9 ± 36.3	0.29
Indexed LGE mass, g/m^2^	7.3 (3.5–15.8)	8.4 (3.9–15.6)	0.98
Indexed LGE volume, ml/m^2^	6.9 (3.3–15.1)	8.0 (3.7–14.9)	0.97
LGE %, % of LV mass	7.9 (4.1–17.9)	8.3 (5.5–14.8)	0.77

Values are presented as percentages, mean ± SD, or median (interquartile range), when appropriate

*RVEF* right ventricular ejection fraction, *IVST* interventricular septal thickness, *LGE* Late Gadolinium Enhancement

### Follow-up data

The follow-up data are shown in Table 4. The percentage of New York Heart Association class III or IV did not differ between baseline and at the last follow-up. At baseline, the proportions of NSVT and left atrium diameter ≥ 45 mm were higher in patients with HOCM and T2DM. However, the prevalence of NSVT remained high in these patients after surgery, whereas left atrium diameter ≥ 45 mm had no difference after surgery. Importantly, the incidence of pulmonary hypertension did not differ preoperatively, but increased significantly during follow-up in patients with T2DM. In addition, patients with HOCM and T2DM had a higher LVOT gradient at baseline.

**Table 4:** Baseline and last follow-up data

Variables	No diabetes (n = 134)	Diabetes (n = 67)	p value
NYHA class III or IV, n			
Baseline	104 (77.6%)	54 (80.6%)	0.63
Last follow-up	6 (4.5%)	7 (10.4%)	0.11
Pulmonary hypertension, n			
Baseline	17 (12.7%)	5 (7.5%)	0.26
Last follow-up	3 (2.2%)	7 (10.4%)	0.02
NSVT, n			
Baseline	15 (11.2%)	15 (22.4%)	0.04
Last follow-up	2 (1.5%)	5 (7.5%)	0.04
LVOT gradient, mmHg			
Baseline	83.3 ± 26.3	93.2 ± 36.8	0.03
Last follow-up	11.6 ± 8.4	12.2 ± 8.5	0.64
Left atrium ≥ 45 mm, n			
Baseline	47 (35.1%)	33 (49.3%)	0.05
Last follow-up	37 (27.6%)	17 (25.4%)	0.74
Death, n	4 (3.0%)	5 (7.5%)	0.16
Sudden cardiac death, n	0 (0%)	3 (4.5%)	0.04

Values are presented as percentage, mean ± SD, or median (interquartile range) when appropriate

*NYHA* New York Heart Association, *NSVT* non-sustained ventricular tachycardia, *LVOT* left ventricular outflow tract, *LVEDD* left ventricular end diastole diameter

### Outcomes and mortality

**Fig. 1:**
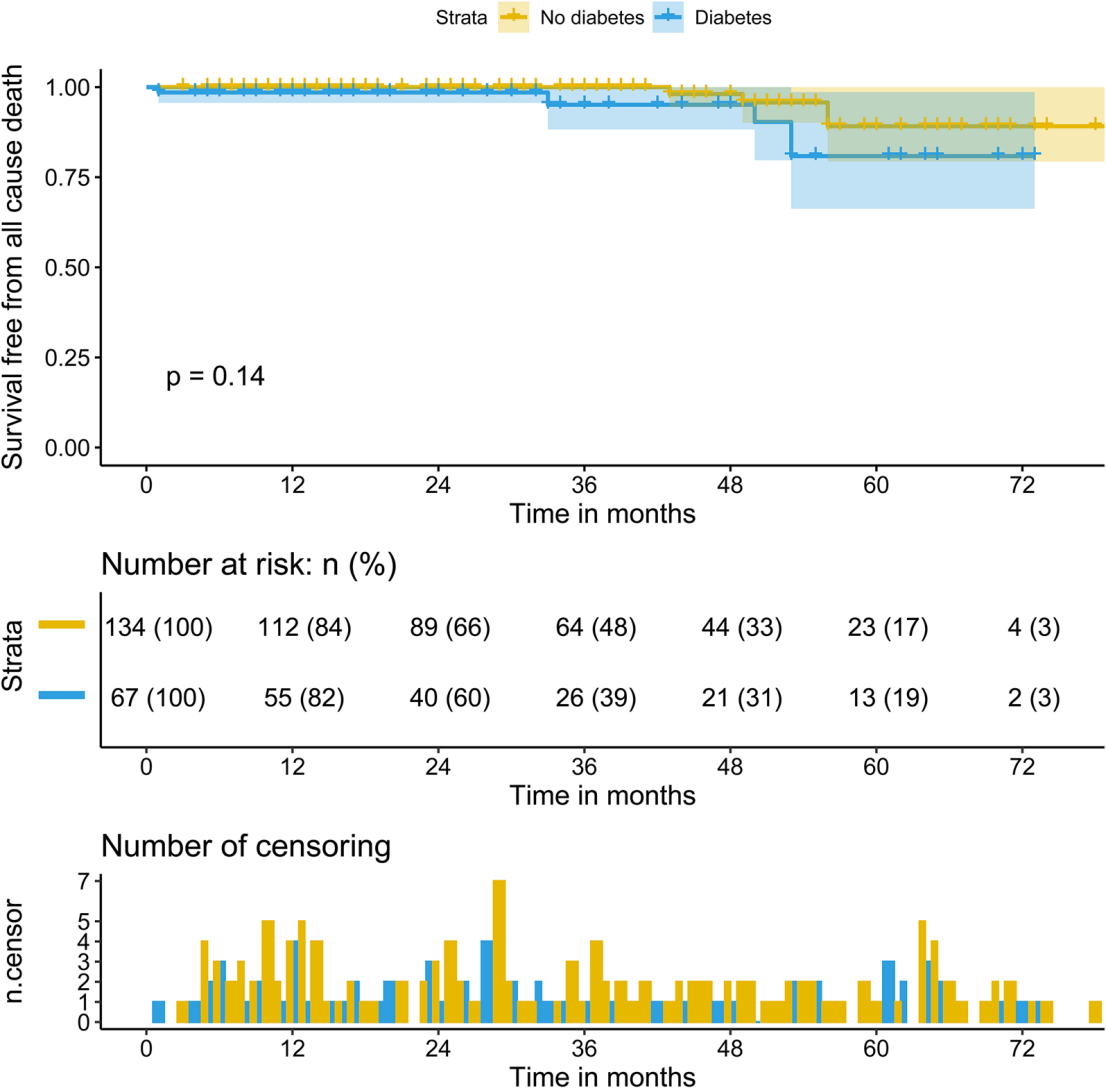
Kaplan-Meier analysis of survival free from cardiovascular death between two groups

**Fig. 2:**
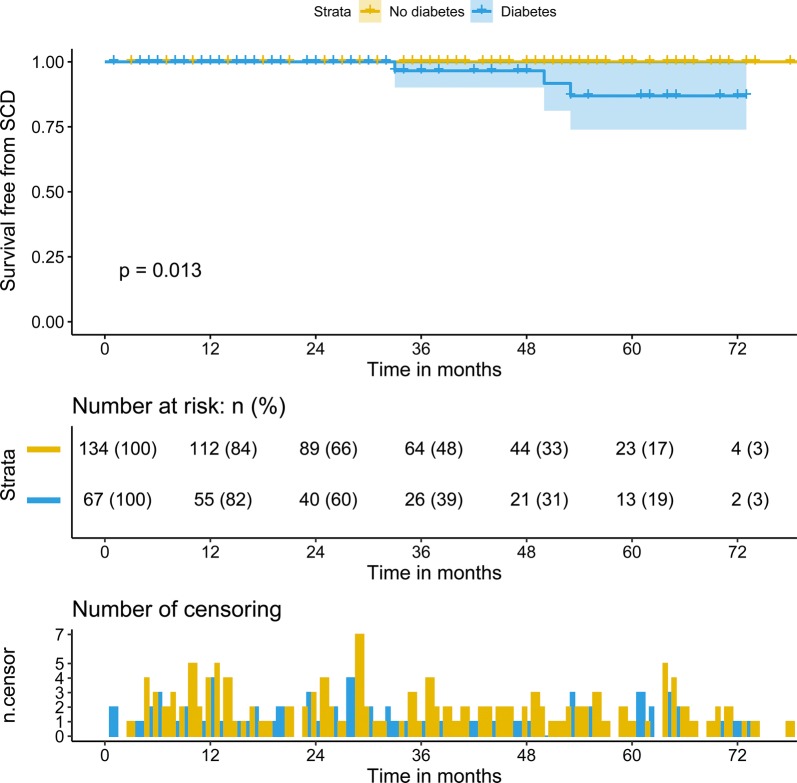
Kaplan-Meier analysis of survival free from SCD between two groups. *SCD* sudden cardiac death

During a median follow-up of 28.0 (IQR: 13.0–53.0) months, 9 patients died. In this study cohort, the cause of death in these patients was cardiovascular death, including 3 SCD, 4 deaths related to heart failure, 1 death due to infective endocarditis, and 1 death due to myocardial infarction. Patients with diabetes had a significantly high rate of SCD (4.5% vs. 0.0%, p = 0.04). The Kaplan–Meier survival analysis revealed no difference in the rates of predicted 3-year survival free from cardiovascular death (98.1% vs. 95.1%, p = 0.14) (Fig. 1). However, the rate of predicted 3-year survival free from SCD (100% vs 96.7%, p = 0.013) was lower in patients with HOCM and T2DM than in those without T2DM (Fig. 2). Furthermore, we analyzed the factors associated with cardiovascular death in patients with HOCM and T2DM. The univariable analysis revealed that history of NSVT (hazards ratio [HR]: 1.51, 95% confidence interval [CI]: 1.31–1.73, p = 0.03), glomerular filtration rate ≤ 80 ml/min (HR:4.84, 95% CI 1.42–19.36, p = 0.02), N-terminal pro-brain natriuretic peptide (NT-proBNP; HR: 1.001, 95% CI 1.000–1.002, p = 0.002), and postoperative LVOT gradient (HR: 1.80, 95% CI 1.08–3.02, p = 0.02) were associated with cardiovascular death in this particular cohort. However, after adjustment for age, and sex, only NT-proBNP (HR: 1.002, 95% CI 1.000–1.005, p = 0.02) and the glomerular filtration rate ≤ 80 ml/min (HR: 3.23, 95% CI 1.34–7.24, p = 0.047) were independent risk factors for patients with T2DM (Table 5).

**Table 5:** Multivariate cox proportional Hazards Models for cardiovascular death in patients with T2DM

Variable	Univariable		Multivariate	
	HR (95% CI)	p	HR (95% CI)	p
Age	0.97 (0.91–1.04)	0.38		
Male	0.49 (0.08–2.96)	0.44		
NT-Pro BNP	1.001 (1.000–1.002)	0.002	1.002 (1.000–1.005)	0.02
eGFR ≤ 80 ml/min	4.83 (1.42–19.36)	0.02	3.23 (1.34–7.24)	0.04
NSVT	1.51 (1.31–1.73)	0.03		
Post-operative LVOT gradient	1.80 (1.08–3.02)	0.02		

*HR* hazards ratio, *eGFR e*stimated glomerular filtration rate, *NSVT* non-sustained ventricular tachycardia, *LVOT* left ventricular outflow tract gradient

## Discussion

Our study investigated the impact of T2DM on the midterm mortality of HOCM patients after septal myectomy. Several findings were included in the present study. First, HOCM patients with T2DM are more likely to develop pulmonary hypertension after surgery, and they have a higher incidence of NSVT even after septal myectomy. Second, HCM patients with and without T2DM have similar 5-year cardiovascular mortality after septal myectomy, but it is associated with higher SCD rate after surgery. Lastly, the baseline NT-proBNP and glomerular filtration rate were independent risk factors among HCM patients with T2DM.

Only a few studies have been reported the impact of T2DM on the clinical outcomes of HCM patients. Recently, one study reported the impact of DM on the clinical phenotype of HCM [3]. They showed that HCM patients with diabetes have a higher cardiovascular risk profile, a lower functional capacity, and more heart failure symptoms. In addition, they found that no difference in the rates of SCD in patients with or without DM. Actually, for patients who underwent septal myectomy have a lower mortality during perioperative period or a long-term result [13, 14]. No study has reported on the influence of T2DM on HCM patients who underwent septal myectomy. However, the impact of DM on the clinical outcomes has been well studied in patients who underwent valve operations and coronary artery bypass. Many studies have reported that DM is associated with significantly worse outcomes after valve operations, and it is an independent predictor for long-term mortality after isolated aortic valve replacement [5, 7, 15]. In addition, previous studies have revealed that DM can increase the incidence of perioperative complications and heart failure and is an independent predictor of long-term mortality after coronary artery bypass [6, 16, 17].

DM can affect the cardiovascular system of HCM patients through many aspects. First, chronic inflammation and microvascular changes within the kidney caused by DM can increase serum levels of inflammatory cytokines and impairment of renal function [18, 19]. In our study, we found that the level of hs-CRP was high and the glomerular filtration rate was low in HCM patients with T2DM. Second, potential contributors of DM to the induction of cardiac arrhythmias, including hyperglycemia or glucose fluctuations and autonomic dysfunction, activate multiple mechanisms that contribute to the development of cardiac arrhythmias. In addition, structural remodeling, including changes in the electrical conduction of the heart, and fibrosis promote and potentiate the progression of arrhythmia [20]. The main finding of our study, i.e., the incidence rates of NSVT and AF were significantly higher in HOCM patients with T2DM than in those without T2DM, was consistent with those of previous studies. Both of these arrhythmias are associated with worse clinical outcomes for HOCM patients. Third, in our study, we found that the prevalence of pulmonary hypertension had no difference before operation, whereas the incidence of pulmonary hypertension after surgery increased significantly. Insulin resistance in DM patients may be a risk factor for pulmonary hypertension [21]. Moreover, in our study, we also found that RVEF was also high in HOCM patients with DM, which may reflect a decrease in right ventricular function. Lastly, the proportion of coronary artery bypass was high in HOCM patients DM. Chronic inflammation, insulin resistance, and dyslipidemia in DM can promote the development of coronary artery disease.

In our study, we found that the rates of predicted 3-year survival free from cardiovascular death are not different between HOCM patients with and without DM after septal myectomy, whereas the rate of predicted 3-year survival free from SCD was significantly lower in patients without DM than in those with DM. This is inconsistent with the previous study that HCM patients with diabetes have a higher risk of cardiovascular mortality and that there was no difference in SCD between patients with and without DM. The reason for this is that none of the patients underwent septal myectomy, and most HCM patients have no LVOT obstruction in that study. The incidence of NSVT was high in HOCM patients with DM even after surgery, which is a known risk factor for SCD [22, 23]. In addition, we analyzed the variables associated with cardiovascular death in the subgroup of HOCM patients with DM. After adjustment for age, sex, and BMI, NT-proBNP and glomerular filtration rate were independent risk factors for cardiovascular death in this particular cohort. This is consistent with the previous studies that the NT-proBNP has been demonstrated as a novel marker for adverse clinical outcomes in HCM patients [24, 25]. Moreover, a previous study revealed that percutaneous transluminal septal myocardial ablation could improve the renal function of patients with HCM, which suggests that these patients may have renal dysfunction [26]. In addition, DM can affect renal function through many ways [19].

DM is the main cause of heart failure, either secondary to cardiovascular disease or secondary to diabetic cardiomyopathy. HCM patients with DM have lower functional capacity and more heart failure symptoms. Therefore, in clinical practice, careful attention should be given for these particular patients who underwent septal myectomy, and they should be need to be followed up more closely, including monitoring of the effects of diabetes on other systems. In addition, because these patients have a relatively higher rate of SCD, we believe that ICD should be implanted more actively in patients with cardiac arrhythmia. By strengthening the management of these patients, we can reduce the complications and improve the prognosis of these patients.

### Limitations

The present study has some limitations. First, this study is limited by its observational nature and the inherent limitations of a retrospective database study. Second, not all patients in our study underwent CMR, which limited our systematic analysis of myocardial fibrosis and right ventricular function in all patients. Third, although the patients in our study were from two medical centers, the number of HOCM patients with DM after septal myectomy is still small. Because of the small number of this particular cohort, we cannot analyze the difference in survival between DM patients who received medication and insulin.

## Conclusions

T2DM is associated with a higher SCD rate in a matched cohort with HOCM who underwent septal myectomy. However, it has no influence on the rate of cardiovascular death of these patients. In addition, the multivariable analysis revealed that the NT-proBNP and glomerular filtration rate were independent risk factors among HOCM patients with T2DM after surgery. The results of the present study suggest that HOCM patients with T2DM should be carefully monitored, especially its effects on renal function, after surgery.

## Supplementary information

**Additional file 1: Table S1.** Relative multivariable imbalance L1 and Summary of unbalanced covariates.

**Additional file 2: Figure S1.** Standardized difference before and after matching.

**Additional file 3: Table S2.** Baseline patient characters in unmatched cohort.

## Data Availability

According to the Fuwai Hospital and Anzhen Hospital system, we are not allowed to share original study data publicly. Therefore, the datasets generated and/or analyzed during the current study are not publicly available.

## Abbreviations

BMI: Body mass index
CI: Confidence interval
CMR: Cardiovascular magnetic resonance
CVD: Cardiovascular death
HCM: Hypertrophic cardiomyopathy
HOCM: Hypertrophic obstructive cardiomyopathy
HR: Hazards ratio
hs-CRP: High-sensitivity C-reactive protein
LGE: Late gadolinium enhancement
LV: Left ventricular
LVOT: Left ventricular outflow tract
NSVT: Non-sustained ventricular tachycardia
NT-proBNP: N-terminal pro-brain natriuretic peptide
SCD: Sudden cardiac death
T2DM: Type 2 diabetes mellitus
