# Supplementary material for: Impact of type 2 diabetes mellitus on mid-term mortality for hypertrophic cardiomyopathy patients who underwent septal myectomy
Source: Cardiovasc Diabetol. 2020 May 13;19:64. doi: 10.1186/s12933-020-01036-1 (PMC7222568; doi:10.1186/s12933-020-01036-1)
Supplement: Supplementary file 1 — Additional file 1: Table S1. Relative multivariable imbalance L1 and Summary of unbalanced covariates. [file 12933_2020_1036_MOESM1_ESM.docx]

Additional file Table S1 Relative multivariable imbalance L1 and Summary of unbalanced covariates

| **Relative multivariable imbalance L1 (lacus, King, & Porro,2010)** | | |
| --- | --- | --- |
|  | Before matching | After matching |
| (all cases) | 0.836 | 0.657 |
| Summary of unbalanced covariates (\|d\|>0.25) | No covariate exhibits a large imbalance (\|d\|>0.25) | |
