# Supplementary material for: Impact of type 2 diabetes mellitus on mid-term mortality for hypertrophic cardiomyopathy patients who underwent septal myectomy
Source: Cardiovasc Diabetol. 2020 May 13;19:64. doi: 10.1186/s12933-020-01036-1 (PMC7222568; doi:10.1186/s12933-020-01036-1)
Supplement: Supplementary file 3 — Additional file 3: Table S2. Baseline patient characters in unmatched cohort. [file 12933_2020_1036_MOESM3_ESM.docx]

Additional file 3: Table S2 Baseline patient characters in unmatched cohort

| Variable | No Diabetes  (n=845) | Diabetes  (n=67) | p Value |
| --- | --- | --- | --- |
| Age, years | 45.7±13.7 | 50.1±13.8 | 0.01 |
| Male, n | 522 (61.8%) | 41 (61.2%) | 0.93 |
| Body mass index, kg/m^2^ | 24.9±3.7 | 25.6±3.7 | 0.18 |
| Family history of HCM or SCD, n | 132 (15.6%) | 15 (22.4%) | 0.15 |
| Heart rate, beats/min | 72.7±9.6 | 72.7±8.5 | 0.98 |
| BNP, pg/mL | 1430.0 (674.6-2669.2) | 1605.5 (573.7-2726.3) | 0.92 |
| Creatinine, umol/L | 75.8±15.2 | 76.8±14.8 | 0.61 |
| Glomerular filtration rate, ml/min | 99.3±21.4 | 98.6±20.7 | 0.78 |
| Hs-CRP, mg/L | 1.03 (0.5-1.9) | 1.30 (0.56-2.28) | 0.08 |
| LDL, mmol/L | 2.6±0.8 | 2.5±0.6 | 0.13 |
| HDL, mmol/L | 1.2±0.4 | 1.16±0.3 | 0.82 |
| Comorbidities |  |  |  |
| Hypertension, n | 172 (20.4%) | 28 (41.8%) | <0.001 |
| Hyperlipemia, n | 103 (12.2%) | 15 (22.4%) | 0.02 |
| Clinical presentation |  |  |  |
| Chest pain, n | 226 (26.7%) | 20 (29.9%) | 0.58 |
| Palpitation, n | 104 (12.3%) | 8 (11.9%) | 0.93 |
| Syncope, n | 112 (13.3%) | 8 (11.9%) | 0.76 |
| Atrial fibrillation, n | 165 (19.5%) | 19 (28.4%) | 0.08 |
| NSVT | 137 (16.2%) | 15 (22.4%) | 0.19 |
| Echocardiographic indices |  |  |  |
| LVEDD, mm | 42.1±5.4 | 41.5±4.5 | 0.39 |
| IVST, mm | 20.4±5.5 | 20.0±5.8 | 0.57 |
| Posterior wall, mm | 12.0±2.7 | 12.2±2.9 | 0.54 |
| LVEF, % | 71.5±6.3 | 71.3±5.4 | 0.83 |
| IVST≥30mm, n | 103 (12.2%) | 4 (6.0%) | 0.13 |
| Left atrial>45mm, n | 403 (47.7%) | 33 (49.3%) | 0.81 |
| LVOT gradient, mmHg | 79.9±26.9 | 93.2±36.8 | 0.005 |
| Moderate or severe MR | 131 (15.5%) | 7 (10.4%) | 0.27 |
| Medical therapy |  |  |  |
| Beta-blockers, n | 615 (72.8%) | 50 (74.6%) | 0.74 |
| Calcium-channel blockers, n | 71 (8.4%) | 7 (10.4%) | 0.56 |
| CABG, n | 53 (6.3%) | 8 (11.9%) | 0.10 |

Values are presented as percentage, mean ± SD, or median (interquartile range) when appropriate.

IVST=interventricular septal thickness; HCM=hypertrophic myocardiopathy; SCD=sudden cardiac death; NYHA=New York Heart Association; BNP=brain natriuretic peptide; LVEF=left ventricular ejection fraction; LDL=low density lipoprotein; HDL=high density lipoprotein; LVEDD=left ventricular end diastole diameter; LVOT =left ventricular outflow tract; MR=mitral regurgitation; ACEI/ARB= angiotensin-converting enzyme inhibitor or angiotensin receptor blocker; CABG= coronary artery bypass graft.
